# Mild Cognitive Impairment and Donepezil Impact Mitochondrial Respiratory Capacity in Skeletal Muscle

**DOI:** 10.1093/function/zqab045

**Published:** 2021-09-02

**Authors:** Jill K Morris, Colin S McCoin, Kelly N Fuller, Casey S John, Heather M Wilkins, Zachary D Green, Xiaowan Wang, Palash Sharma, Jeffrey M Burns, Eric D Vidoni, Jonathan D Mahnken, Kartik Shankar, Russell H Swerdlow, John P Thyfault

**Affiliations:** Department of Neurology, University of Kansas Medical Center, Kansas City, KS, USA; University of Kansas Alzheimer's Disease Center, Kansas City, KS, USA; Department of Molecular and Integrative Physiology and Internal Medicine-Division of Endocrinology and Metabolism, University of Kansas Medical Center, Kansas City, KS, USA; Department of Molecular and Integrative Physiology and Internal Medicine-Division of Endocrinology and Metabolism, University of Kansas Medical Center, Kansas City, KS, USA; Department of Neurology, University of Kansas Medical Center, Kansas City, KS, USA; University of Kansas Alzheimer's Disease Center, Kansas City, KS, USA; Department of Neurology, University of Kansas Medical Center, Kansas City, KS, USA; University of Kansas Alzheimer's Disease Center, Kansas City, KS, USA; Department of Neurology, University of Kansas Medical Center, Kansas City, KS, USA; University of Kansas Alzheimer's Disease Center, Kansas City, KS, USA; Department of Neurology, University of Kansas Medical Center, Kansas City, KS, USA; University of Kansas Alzheimer's Disease Center, Kansas City, KS, USA; Department of Biostatistics, University of Kansas Medical Center, Kansas City, KS, USA; Department of Neurology, University of Kansas Medical Center, Kansas City, KS, USA; University of Kansas Alzheimer's Disease Center, Kansas City, KS, USA; Department of Neurology, University of Kansas Medical Center, Kansas City, KS, USA; University of Kansas Alzheimer's Disease Center, Kansas City, KS, USA; Department of Biostatistics, University of Kansas Medical Center, Kansas City, KS, USA; University of Kansas Alzheimer's Disease Center, Kansas City, KS, USA; Pediatrics, Section of Nutrition, The University of Colorado Anschutz Medical Campus, Aurora, CO, USA; University of Kansas Alzheimer's Disease Center, Kansas City, KS, USA; Department of Neurology, University of Kansas Medical Center, Kansas City, KS, USA; University of Kansas Alzheimer's Disease Center, Kansas City, KS, USA; Department of Molecular and Integrative Physiology and Internal Medicine-Division of Endocrinology and Metabolism, University of Kansas Medical Center, Kansas City, KS, USA; Department of Neurology, University of Kansas Medical Center, Kansas City, KS, USA; Research Service, Kansas City VA Medical Center, Kansas City, MO, USA

**Keywords:** mitochondria, mild cognitive impairment, Alzheimer's disease, skeletal muscle, respiration, RNA sequencing, donepezil

## Abstract

Alzheimer's Disease (ad) associates with insulin resistance and low aerobic capacity, suggestive of impaired skeletal muscle mitochondrial function. However, this has not been directly measured in AD. This study ( *n*  = 50) compared muscle mitochondrial respiratory function and gene expression profiling in cognitively healthy older adults (CH; *n* = 24) to 26 individuals in the earliest phase of ad-related cognitive decline, mild cognitive impairment (MCI; *n*  = 11) or MCI taking the ad medication donepezil (MCI + med; *n*  = 15). Mitochondrial respiratory kinetics were measured in permeabilized muscle fibers from muscle biopsies of the vastus lateralis. Untreated MCI exhibited lower lipid-stimulated skeletal muscle mitochondrial respiration (State 3, ADP-stimulated) than both CH ( *P* = .043) and MCI + med (*P* = .007) groups. MCI also exhibited poorer mitochondrial coupling control compared to CH (*P* = .014). RNA sequencing of skeletal muscle revealed unique differences in mitochondrial function and metabolism genes based on both MCI status (CH vs MCI) and medication treatment (MCI vs MCI + med). MCI + med modified over 600 skeletal muscle genes compared to MCI suggesting donepezil powerfully impacts the transcriptional profile of muscle. Overall, skeletal muscle mitochondrial respiration is altered in untreated MCI but normalized in donepezil-treated MCI participants while leak control is impaired regardless of medication status. These results provide evidence that mitochondrial changes occur in the early stages of AD, but are influenced by a common ad medicine. Further study of mitochondrial bioenergetics and the influence of transcriptional regulation in early ad is warranted.

## Graphical Abstract

**Graphical Abstract:**
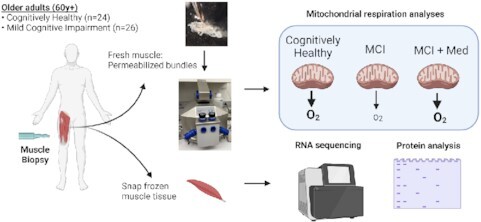

## Introduction

Emerging evidence suggests that altered energy metabolism may play a role in the development and progression of Alzheimer's Disease (ad). Results show that mitochondrial function is compromised systemically in ad,^1^ ranging from platelets to brain, and we have shown that ad subjects have both reduced aerobic capacity/cardiorespiratory fitness^2^ and reduced insulin sensitivity^3^ compared to cognitively healthy older adults. Skeletal muscle mitochondrial dysfunction is linked to whole body insulin resistance,^4–6^ a recognized risk factor for AD.^7–19^ Moreover, skeletal muscle mitochondrial content and functional capacity plays a key role in maximal aerobic capacity. Given that skeletal muscle tissue comprises 30–40% of body mass and declines with aging,^20^ metabolic deficits in muscle have a profound impact on systemic metabolism and development of age-related chronic disease states. However, skeletal muscle mitochondrial function has never been directly assessed in cognitively impaired individuals.

The overall goal of this study was to test the hypothesis that individuals in the earliest stage of ad-related cognitive decline, Mild Cognitive Impairment (MCI), possess reduced mitochondrial function and specifically, reduced State 3 (ADP-stimulated) skeletal muscle mitochondrial respiratory capacity compared to cognitively healthy elderly adults (CH). We also postulated that differences in respiratory capacity could be driven by ad genetic risk (Apolipoprotein ε4; *APOE4*) or non-genetic risk (overweight/obesity, physical activity, or other factors). Emerging literature also suggests that the ad medication donepezil influences mitochondrial function in various model systems.^21–23^ Because over half (58%) of MCI subjects in our study were actively using AD medication, we sought to characterizeskeletal muscle mitochondrial effects in the presence and absence of donepezil treatment Our results show a clear effect of MCI status being linked to compromised mitochondrial function in skeletal muscle that is partially modified by medication status.

## Methods

### Ethical Approval and Recruitment

This study was approved by the University of Kansas Medical Center's Institutional Review Board (IRB number 140 787). All participants in this study provided informed consent according to institutional guidelines and in accordance with the Declaration of Helsinki. Participants were recruited by the KU Alzheimer's Disease Center (KU ADC) recruitment division as previously described.^24^ 180 qualifying individuals were prescreened for this study by the KU ADC recruitment operations team. A total of 58 individuals were selected for additional in-depth screening by the study coordinator, with 52 participants enrolled in the study. In total, one individual was withdrawn from the study prior to the muscle biopsy due to safety labs out of range and one individual was excluded from analysis due to the muscle sample being compromised prior to analysis. We thus report results from 50 participants.

All individuals were over 60 yr of age and on stable medication doses for at least 30 d. Diagnostic inclusion criteria included no prior memory complaints (CH older adults), or MCI diagnosed by a clinician and verified with medical records. Individuals were excluded from participating if they had moderate or severe ad, other neurological disorders that could impair cognition, evidence of bleeding disorders during screening (elevated PT/PTT values), prior bleeding problems or use of antiplatelet medication, Warfarin, or any other anticoagulant, clinically significant disease, clinically significant psychiatric disorder, systemic illness, or infection likely to affect safety, clinically-evident stroke, myocardial infarction or coronary artery disease in the last 2 yr, insulin dependent diabetes, or significant pain or musculoskeletal symptoms that would affect safety. In total, one individual in the CH group had a diagnosis of Type 2 diabetes but was not using diabetic medication.

Interested participants completed a brief phone screen to verify safety to complete visits. Participants that appeared eligible were scheduled an in-person visit. A screening blood panel was also performed prior to the muscle biopsy to verify that no blooding disorders were present. Individuals completed two study visits: Visit 1 (Graded exercise test; GXT, and dual energy x-ray absorptiometry (DXA) scan), and Visit 2 (fasting blood draw and muscle biopsy).

### Medications

Medication use was verified by medical record. All individuals in the study were required to be on stable medication doses for greater than 30 d prior to enrollment. A total of 15 MCI subjects (58% of study subjects with MCI) were routinely taking medication for mild memory complaints. All 15 of these individuals were using the cholinesterase inhibitor donepezil (10 mg/d;  12 or 5 mg/d;  3,  due to inability to tolerate a 10 mg/d dose). Of these 15 subjects, five individuals were also taking memantine, an NMDA receptor antagonist (5–28 mg/d).

### Anthropometric Measures

Individuals reported to the KU Clinical and Translational Research Unit (CTSU) for Visit 1 following an overnight fast. After a 5 min rest period, vital signs were obtained. We measured height in cm and total body mass using a digital scale accurate to 0.1 kg (Seca Platform Scale, model 707). Waist and hip circumference were also measured. Subjects were asked to void and evaluated using a DXA scan (Lunar Prodigy, version 11.2068) to determine lean mass, fat mass, and bone mineral density.

### Cardiorespiratory Fitness Testing

A GXT was performed according to a modified Bruce protocol on a treadmill as previously described to determine cardiorespiratory fitness.^25^ Subjects were attached to a 12-lead electrocardiograph to monitor cardiac rhythm, and a non-rebreathing mask to continuously capture oxygen and carbon dioxide for 3 min while seated and relaxed at rest. This allowed for calculation of a resting Respiratory Exchange Ratio (RER). To begin the test, each participant mounted a treadmill and began walking with speed and incline gradually increasing with each 2 min stage as previously reported.^26^ Blood pressure and rating of perceived exertion were collected at the end of each stage. The test was terminated if the participant reached volitional fatigue or met the absolute test termination criteria (RER> = 1.1, RPE 17, plateau in VO_2_/100 mL change, 90% HRmax). Maximal oxygen uptake during the GXT was calculated relative to whole body mass (mL/kg/min). Our group has previously validated VO_2_ peak testing in both AD subjects and in CH older adults.^25,27,28^

### Accelerometry

At Visit 1, individuals were given a wrist-worn Actigraph GT9X watch (Sample Rate 30Hz) and asked to wear the watch for 7 d to capture both weekday and weekend physical activity and sedentary behavior patterns. Accelerometers were returned to the study team during Visit 2 for data analysis. Wear validation was performed using a computer algorithm^29^ with a minimum of 600 mins wear time per day and 4 valid days required for inclusion of data. No individuals were excluded from analysis due to inadequate wear time. In total, one subject was excluded from the analyses due to corrupt data. Analyses were performed using Actigraph ActiLife 6 software (v6.13.3).

### Blood Biomarkers

Approximately 2 wk following Visit 1, subjects returned to the KU Clinical Translational Science Unit (CTSU) for visit 2 following an overnight fast. Blood samples were analyzed using a lipid panel (Quest Diagnostics) to determine cholesterol HDL, LDL, LDL/HDL, and triglyceride values. Blood was collected into serum separator tubes and processed to generate plasma and serum, which was frozen at −80°C until further analysis. Glucose was measured using a colormetric assay (Sigma-Aldrich, St. Louis, MO) and insulin was measured using ELISA (Alpco Diagnostics). Amyloid beta 42 (Aβ42), Amyloid beta 40 (Aβ40), neurofilament light (NFL), and glial fibrillary acidic protein (GFAP) were measured on a Simoa HD-X machine in plasma using the Neurology 4-Plex E kit (Quanterix).

### Muscle Biopsy and Mitochondrial Respirometry Analyses

Following the visit 2 blood draw, a skeletal muscle biopsy of the vastus lateralis (∼120 mg tissue) was performed. Freshly obtained muscle tissue was immediately dissected free of connective tissue and separated into segments, with a portion (∼30 mg) placed into ice cold buffer X (50 mM K-MES, 7.23 mM K_2_EGTA, 2.77 mM CaK_2_EGTA, 20 mM Imidazole, 20 mM Taurine, 5.7 mM Na-ATP, 14.3 mM Na-PCr, and 6.56 mM MgCl_2_-6H_2_O; pH 7.1) for transport to the laboratory and analysis of respiratory kinetics.

Basal and ADP-stimulated respiratory kinetics was measured in parallel in the presence of malate and palmitoylcarnitine (lipid) or pyruvate (carbohydrate) in duplicate on permeabilized fiber bundles in an Oroboros Oxygraph-2k system (Innsbruck, Austria). All mitochondrial analyses were completed in real time within 2–3 h of the biopsy. Multiple small fiber bundles were teased from the ∼30 mg muscle sample on ice using fine forceps under a dissecting scope in ice cold buffer X. Following fiber bundle preparation, bundles were placed into buffer X containing 30 ug/mL saponin for 30 min on a rotator at 4°C, prior to washing with ice cold Buffer Z (105 mM K-MES, 30 mM KCl, 10 mM K_2_HPO_4_, 5 mM MgCl_2_-6H_2_O, and 0.5% w/w fatty acid-free BSA; pH 7.1) + 0.5 M EGTA.

O_2_ consumption rates were quantified with high resolution respirometry within prepared muscle fiber bundles. Analyses were performed at 37°C in Buffer Z with 0.5 M EGTA and 20 mM creatine monohydrate. Basal respiratory kinetics were obtained in the presence of 0.01 mM blebbistatin, 0.02 mM palmitoyl CoA, 0.5 mM malate, 5 mM carnitine, and 0.018 mM palmitoylcarnitine or 5mM pyruvate. O_2_ was added to the chamber for an initial concentration of ∼260 μM. After reaching steady state, this was followed by sequential addition of 4 mM ADP to assess State 3 respiration through Complex I, and 10 mM succinate (Complex II substrate; State 3S). We assessed uncoupled respiration by adding carbonylcyanide-p-trifluoromethoxyphenylhydrazone (FCCP, a protonophoric uncoupler) until we observed maximal respiration. Following analyses, fibers were retrieved from the chamber, briefly rinsed in dH2O, freeze dried overnight in a lyophilizer (FreeZone 2.5 L,  Labconco, Kansas City, MO) and weighed using a microbalance (MX5, Mettler Toledo, Columbus, OH). Mitochondrial respiration values were normalized to dry muscle weight, and are thus expressed as pmol/s/mg dry weight per mL. We calculated coupling control ratio by dividing leak respiration by phosphorylating State 3 respiration (ADP condition; L/P). We calculated leak control ratio by dividing leak respiration by FCCP stimulated respiration (L/E).

### Protein Expression Analyses

Western blotting was performed on a subset of muscle samples (44/50). Frozen muscle was powdered and weighed prior to processing with a TissueLyser II bead homogenizer (Qiagen, Germantown, MD) in buffer containing 50 mM HEPES, 12 mM sodium pyrophosphate, 100 mM NaF, 10 mM EDTA, 400 μL each phosphatase inhibitor cocktail 2 and 3 (Sigma-Aldrich), and 1% Triton X-100. Protein concentration assessed using a Pierce BCA assay (Thermo Scientific, Rockford, IL) and samples prepared for western blotting prior to separation using SDS-PAGE. Proteins were transferred to PVDF membrane prior incubation with Total OXPHOS primary antibody (Abcam, Cambridge, MA). Densitometry was measured using ImageLab 5.2.1 (BioRad, Hercules, CA) and protein loading corrected using 0.1% amido-black (Sigma-Aldrich).

### RNA Sequencing and Analysis

We isolated RNA and performed RNA sequencing on a subset of participants (47/50 total. Isolated RNA from skeletal muscle was cleaned with the QIAamp RNA Blood Mini Kit (52304) according to the standard protocol. The cleaned RNA was quantified using a Qubit RNA BR Assay Kit (ThermoFisher, Q10210) and the RIN was assessed on an RNA ScreenTape (Agilent, Catalog number 5067-5577 and 5067-5576) on the TapeStation platform prior to library preparation. RNA libraries were prepared using a TruSeq Stranded RNA HT Sample Prep Kit (Illumina, Catalog number RS-122–2303) on a Caliper (now Perkin Elmer) Sciclone G3 platform. An additional standard 1.0x manual bead cleanup was performed using AMPure XP Beads (Beckman Coulter, A63881) after the libraries were completed to clean up primer-dimers. Qubit dsDNA BR Assay kit was used to determine the concentration of the completed library and a Fragment Analyzer Standard Sensitivity NGS Fragment Kit (Agilent, Catalog number DNF-473-10 000) was used to detect the size of the library and to verify removal of excess primer-dimers. Standard Illumina Free-Adapter Blocking was performed on the completed libraries. Cleaned, adapter-blocked libraries were loaded on a NovaSeq6000 with a run configuration of 151 × 8 × 8×151 and an average depth of 84M PE reads per library.

Following sequencing and demultiplexing, all reads were trimmed for adapters, filtered based on quality score, and aligned to the human genome (hg19) using the STAR aligner. Resulting read alignments for each sample were imported in Seqmonk for gene level quantification as counts mapping to annotated genes. Gene counts were imported into R (v4.05) and analysis of differential expression between groups was done using the limma-voom pipeline.^30^ Transcripts with expression below 0.5 counts/million were excluded from further analysis. Differentially expressed genes between two pair-wise comparisons (MCI vs HC; and MCI + Med vs MCI) were identified with a nominal *P*-value < .05 and a minimum fold change 1.5 fold. Further interpretive analysis of differentially expressed genes for enrichment of gene ontology (GO) terms was done using Enrichr^31^ and HumanBase functional module detection.^32^ The HumanBase functional module analysis, utilizes tissue-specific network-based functional interpretation of gene lists, and applies community detection to find cohesive gene clusters from a provided gene list (https://hb.flatironinstitute.org/module/). Heatmaps were generated using Morpheus (https://software.broadinstitute.org/morpheus).

## Statistics

Group differences for continuous variables were assessed using ordinary least squares regression to perform ANOVA-like analyses with adjustment for other factors. Significant findings were further explored using LSD posthoc analyses. For an overall assessment of mitochondrial response under multiple substrate conditions, we used linear mixed modeling to account for repeated measures. Model assessment included residual analysis. Linear mixed model results assuming the normal distribution were violated with respect to the constant variance assumption. Log-transformation did not correct this problem with the residuals. We thus adjusted to use a generalized linear mixed model assuming an underlying Poisson distribution as this allowed for the variance to increase as a function of the mean, and this resolved the poor diagnostic indications from the linear mixed models. The relationship between CRF and our primary mitochondrial outcome measure of State 3 (ADP) flux was explored using linear regression. All analyses were adjusted for age, sex, *APOE4* carrier status, and overweight/obesity status. Data presented in Table 1 as mean [SD]. Results were considered significant at *P < .05.

**Table 1.** Participant Characteristics

Outcome	CH (*n* = 24)	MCI (*n* = 11)	MCI + Med (*n* = 15)	*P*-value
**Age (y)**	72.5 [8.5]	69.6 [7.4]	74.9 [7.3]	0.253
**Sex (#, % female)**	12 [48%]	9 [82%]	7 (47%]	0.128
**APOE4 (#, % carrier)**	9 [36%]	8 [73%]	7 [47%]	0.126
**Education (y)**	16.9 [1.7]	16.9 [2.3]	15.8 [2.3]	0.250
**Overweight/Obesity (#,%)**	15 [62%]	3 [27%]	9 [60%]	0.130
**Activity per day (counts)**	12 689.8 [3470]	14 936 [3242]	10 121.0 [3014]	0.010*^,+^
**VO_2 peak_ (mL/kg/min)**	26.5 [6.9]	25.8 [4.2]	22.8 [6.7]	0.155
**GXT duration (min)**	13.1 [2.6]	12.4 [2.5]	9.51 [3.3]	<0.001*^,+^
**Resting RER**	0.831 [0.08]	0.773 [0.04]	0.780 [0.08]	**0.031^#,+^**
**Peak HR**	159.8 [20.1]	154.9 [15.0]	148.7 [27.4]	0.437
**BMD**	1.25 [0.19]	1.11 [0.16]	1.20 [0.13]	0.228
**Waist Hip Ratio**	0.883 [0.12]	0.814 [0.08]	0.917 [0.11]	0.276
**Lean mass (kg)**	47.5 [10.7]	37.9 [10.6]	48.3 [10.4]	0.100
**Fat mass (kg)**	27.0 [9.3]	19.5 [5.1]	29.9 [11.2]	**0.027** ^+^

APOE; Apolipoprotein E, BMI; body mass index, GXT; graded exercise test, RER; respiratory exchange ratio, and HR; heart rate. Mean values are given as means [SD] and were assessed using ANCOVA. *P* < .05 ^#^CH vs MCI, ^+^CH vs MCI + med, and *MCI vs MCI + med.

## Results

### Subject Characteristics

CH, MCI, and MCI + med groups did not significantly differ by age, sex, genotype (*APOE4* carrier status), or overweight/obesity status (Table 1). Within the MCI + med group, all participants (*n* = 15, 100%) were using the ad medication donepezil, an acetylcholinesterase inhibitor. Of those subjects, 33% (*n* = 5) were also using the ad medication memantine. There was no difference in years of education between groups. Scores for the Mini-Mental State Examination (MMSE) were available for all MCI subjects. MMSE scores were not different between MCI and MCI + med groups (mean MMSE score 26 for each group), and these scores are within the range commonly observed in MCI populations.^33^

All groups were highly active (>10 000 activity counts per day), but differed in physical activity (*P* = .010), with MCI + med subjects exhibiting slightly lower activity counts compared to both CH (*P* = .008) and MCI subjects (*P* = .009; Table 1). This did not translate into aerobic capacity differences; all participants completed a graded exercise text (GXT) and overall fitness levels (VO_2 peak_) were not different between groups. However, the duration of the GXT differed significantly (*P* < .001) with a shorter test duration observed in MCI + med subjects compared to both CH and MCI subjects (CN vs MCI, *P* < .001, MCI vs MCI + med, *P* = .012). RER was measured in the fasted, rested condition prior to the GXT to assess substrate utilization patterns. Resting RER differed between groups (*P* = .031), with lower RER in MCI (*P* = .023) and MCI + med subjects (*P* = .044), suggesting potential differences in resting substrate metabolism. We also observed differences in fat mass (*P* = .027), with MCI individuals exhibiting less fat mass than MCI + med subjects (*P* = .007) and marginally lower fat mass compared to CH individuals (*P* = .060). Lean mass and bone mineral density were not different amongst groups (Table 1).

### Blood Biomarkers

We did not observe group differences for plasma cholesterol, triglycerides, Low Density Lipoprotein (LDL), High Density Lipoprotein (HDL), glucose, or insulin (Table 2). LDL/HDL ratio and homeostatic model assessment of insulin resistance (HOMA-IR) also did not differ between groups. Neurodeneration-related biomarkers were available in 49 subjects. As expected, group differences were observed with plasma glial fibrillary acidic protein (GFAP; *P* = .018), an astrocytic marker of injury, neurofilament light (NFL; *P* = .007), a marker of neurodegeneration, and plasma amyloid beta 42:42 ratio (Aβ42:40), an indicator of ad neuropathology (*P* = .029). Posthoc analyses revealed these differences between CH and both MCI and MCI + med groups, with the exception of GFAP, where there was a trend (*P* = .09) between CH and MCI with significance between CH and MCI + med (*P* = .01; Table 2).

**Table 2.** Metabolic and AD-related Biomarkers

Outcome	CH	MCI	MCI + Med	*P*-value
**Glucose (mg/dL)**	90.3 [9.0]	93.3 [12.6]	97.5 [10.9]	0.181
**Insulin (µU/mL)**	13.4 [30.8]	18.0 [44.3]	10.3 [11.6]	0.830
**Cholesterol (mg/dL)**	186.5 [41.7]	190.1 [38.1]	186.5 [53.6]	0.991
**Triglycerides (mg/dL)**	97.0 [53.2]	85.9 [26.7]	98.0 [51.0]	0.770
**HDL (mg/dL)**	64.8 [19.4]	73.2 [15.6]	59.5 [12.8]	0.663
**LDL (mg/dL)**	102.4 [35.0]	99.8 [35.0]	104.7 [46.8]	0.792
**LDL/HDL (mg/dL)**	1.63 [0.52]	1.42 [0.66]	1.77 [0.80]	0.747
**HOMA-IR (mg/dL)**	3.02 [6.9]	4.37 [10.9]	2.55 [3.0]	0.813
**GFAP (pg/mL)**	167.4 [68.6]	215.2 [75.8]	232.7 [84.9]	**0.018^+^**
**NFL (pg/mL)**	18.9 [7.20]	26.3 [6.69]	25.4 [6.43]	**0.007^#,+^**
**Aβ 42:40**	0.061 [0.009]	0.053 [0.008]	0.053 [0.009]	**0.029^#,+^**

Fasting serum biomarker levels do not differ amongst groups. HDL; high density lipoprotein, LDL; low density lipoprotein; HOMA-IR; homeostasis model assessment of insulin resistance, GFAP; glial fibrillary acidic protein, NFL; neurofilament light; Aβ 42:40; amyloid beta 42:40 ratio. Values are given as means [SD] and were assessed using ANCOVA. *P* < .05 ^#^CH vs MCI, ^+^CH vs MCI + med.

### Mitochondrial Respiration

**Figure 1.**
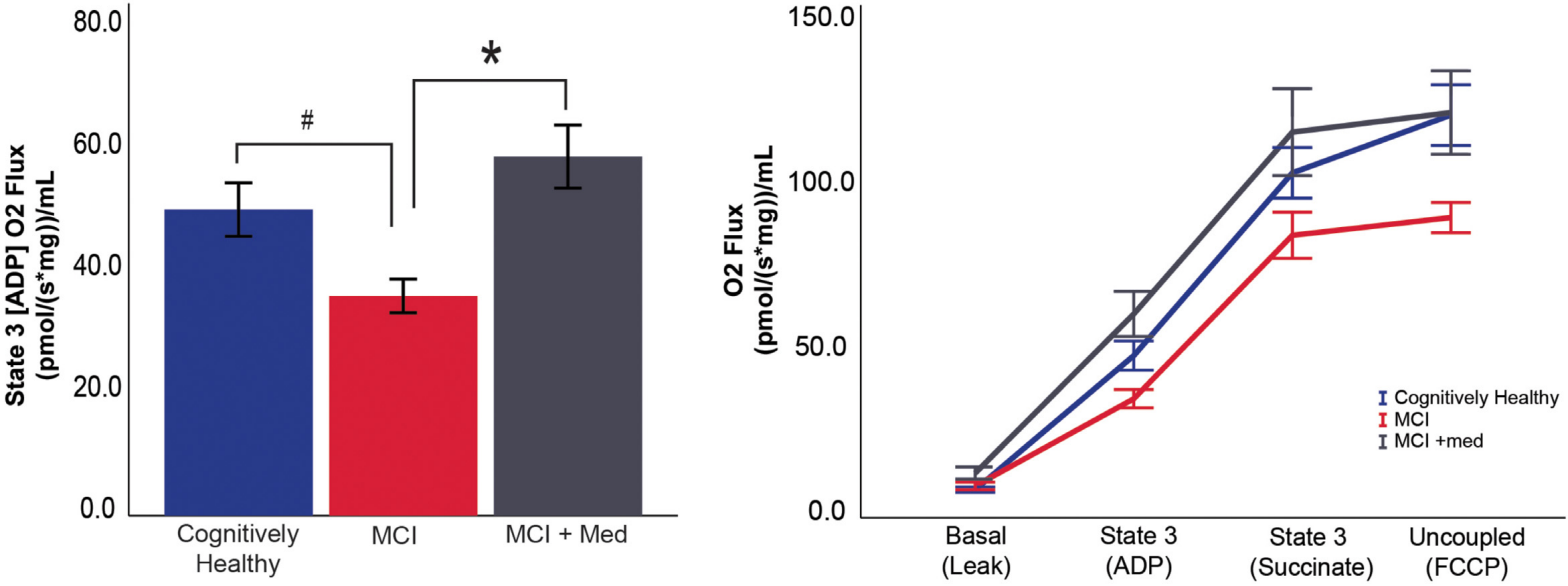
Skeletal muscle mitochondrial respiration differs between groups. Lipid (palmitoylcarnitine) stimulated skeletal muscle mitochondrial respiration was lower in MCI subjects during State 3 (ADP) compared to both other groups (A). Responses were also assessed under a sequence of conditions, and the overall group response differed across the states (*P* < .001). Ordinary least squares regression was used for State 3 univariate analyses. The repeated measures overall responses to substrate conditions was assessed using linear mixed modeling. Both sets of analyses adjusted for covariates. Data represent mean ± SE. #*P* < .05 MCI no drug vs CH. *P < .05 MCI vs MCI + med. CH *n* = 24, MCI *n* = 11, and MCI + med *n* = 15 subjects per group.

To test mitochondrial function, we measured respiratory kinetics in permeabilized skeletal muscle fiber bundles under multiple substrate conditions. Parallel assessments were performed in duplicate in the presence of lipid (palmitoylcarnitine) and carbohydrate (pyruvate). Analysis of our primary outcome measure, the respiratory response to ADP, was different between groups in the lipid-stimulated condition. We observed differences in ADP-stimulated (State 3) respiratory kinetics (*P* = .020; Figure 1A), with MCI subjects exhibiting lower mitochondrial respiration than CH (*P* = .043) and MCI + Med (*P* = .006) individuals. We also performed multiple additional titrations of substrates, including Succinate (State 3S) and FCCP (uncoupled respiration) to assess respiratory control under a sequence of conditions. In our analysis accounting for these repeated measures across all sequence conditions, we also found the relationship between groups differed across the sequence of conditions (*P* < .001). Within condition difference between groups were detected in this repeated measures analysis for the ADP-stimulated (State 3) condition (*P* = .005), and for Basal, Succinate (State 3S), and FCCP group comparison tests resulted in *P* = .067, *P* = .254, and *P* = .073, respectively (Figure 1B). In this repeated measures analysis, within the ADP-stimulated condition MCI subjects again indicated lower mitochondrial respiration than CH (*P* = .016) and MCI + Med (*P* = .001) individuals.

**Figure 2.**
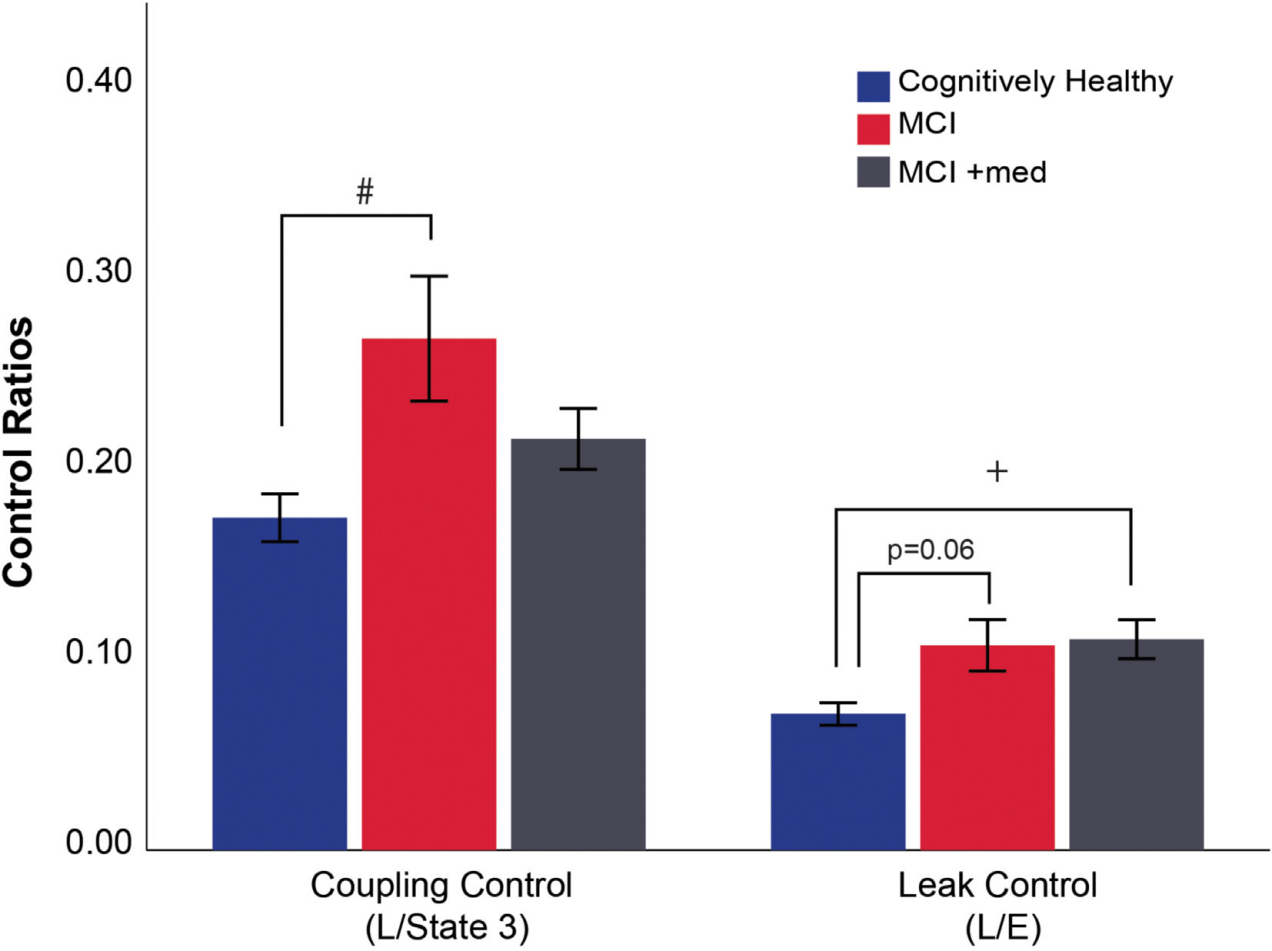
Diagnostic differences in mitochondrial control ratios. MCI subjects not treated with mediation exhibited poorer coupling control (higher control ratio) compared to CH subjects. Both MCI groups exhibited poorer leak control compared to CH individuals. Group differences were assessed using ANCOVA, adjusting for covariates. Data represent mean ± SE. #*P* < .05 CH vs MCI, +*P* < .05 CH vs MCI + med. CH *n* = 24, MCI *n* = 11, and MCI + med *n* = 15 subjects per group.

Coupling control ratio provides information on the coupling of electron flux through the electron transport chain to ATP production (lower values equal higher coupling). Coupling control ratio was significantly different between groups (*P* = .045; Figure 2A). MCI subjects exhibited poorer coupling control compared to CH subjects (*P* = .014), while MCI + med and CH subjects did not differ. Similarly, leak control ratio, which can be used to assess uncoupling during constant electron transport flow was also different between groups (*P* = .019; Figure 2B). Leak control ratio was impaired in the MCI + med group compared to CH subjects (*P* = .008) with a trend for a difference in MCI vs CH (*P* = .06), suggesting greater uncoupling of the system in MCI regardless of medication status. Our identical analyses of respiratory kinetics in the presence of pyruvate did not show significant differences between groups (Figure S1).

### Relationship Between Mitochondrial Respiration and Fitness

**Figure 3.**
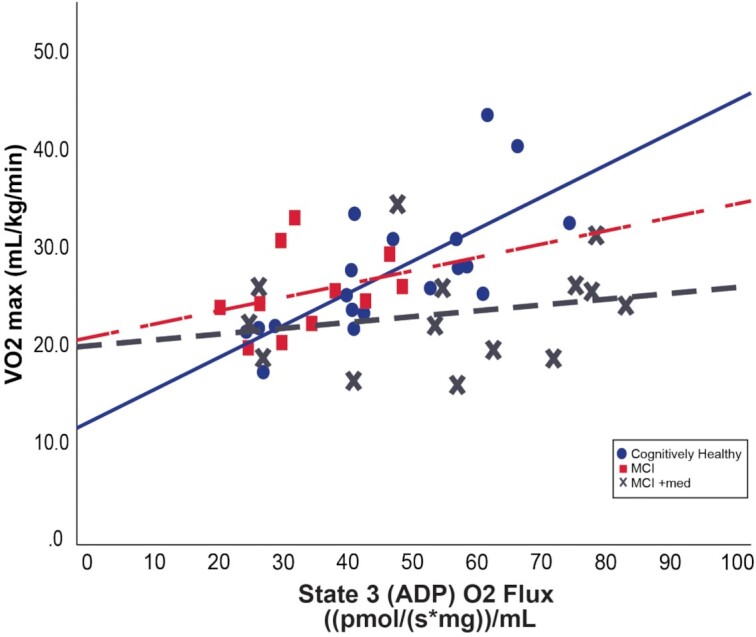
Relationship between skeletal muscle respiration and aerobic capacity. In CH (solid blue line) and MCI (dashed red line) individuals who met established criteria for a reliable graded exercise test (*n* = 44), cardiorespiratory fitness tracks with State 3 (ADP) respiration (CH; β = 0.615, *P* = .024 and MCI; β = 0.615, *P* = .036). There is no significant relationship in medication-treated MCI subjects (dashed gray line). Relationships were assessed within each group using linear regression, adjusting for covariates. CH *n* = 19, MCI *n* = 11, and MCI + med *n* = 14 subjects per group.

We next tested if the observed group differences in State 3 (ADP) respiration were correlated to maximal aerobic capacity (VO_2max_) measured during the GXT. When we examined only individuals who met American College of Sports Medicine (ACSM) criteria for a reliable maximal exercise test (VO_2max_, *n* = 44 subjects) we observed a positive linear relationship between VO_2max_ and State 3 (ADP) skeletal muscle respiration in both CH subjects (β = 0.615, *P* = .024) and MCI individuals (β = 0.615, *P* = .036; Figure 3). There was no significant relationship between VO_2max_ and skeletal muscle respiration in MCI + med participants, suggesting a potential dyscoupling of mitochondrial function and maximal aerobic capacity in those individuals. We did not observe a significant relationship between State 3 (ADP) respiration and maximal aerobic capacity when including all participants regardless of ACSM criteria, indicating that the exercise test quality is an important factor when investigating these relationships.

### Gene and Protein Expression of Electron Transport System Related Factors

**Figure 4.**
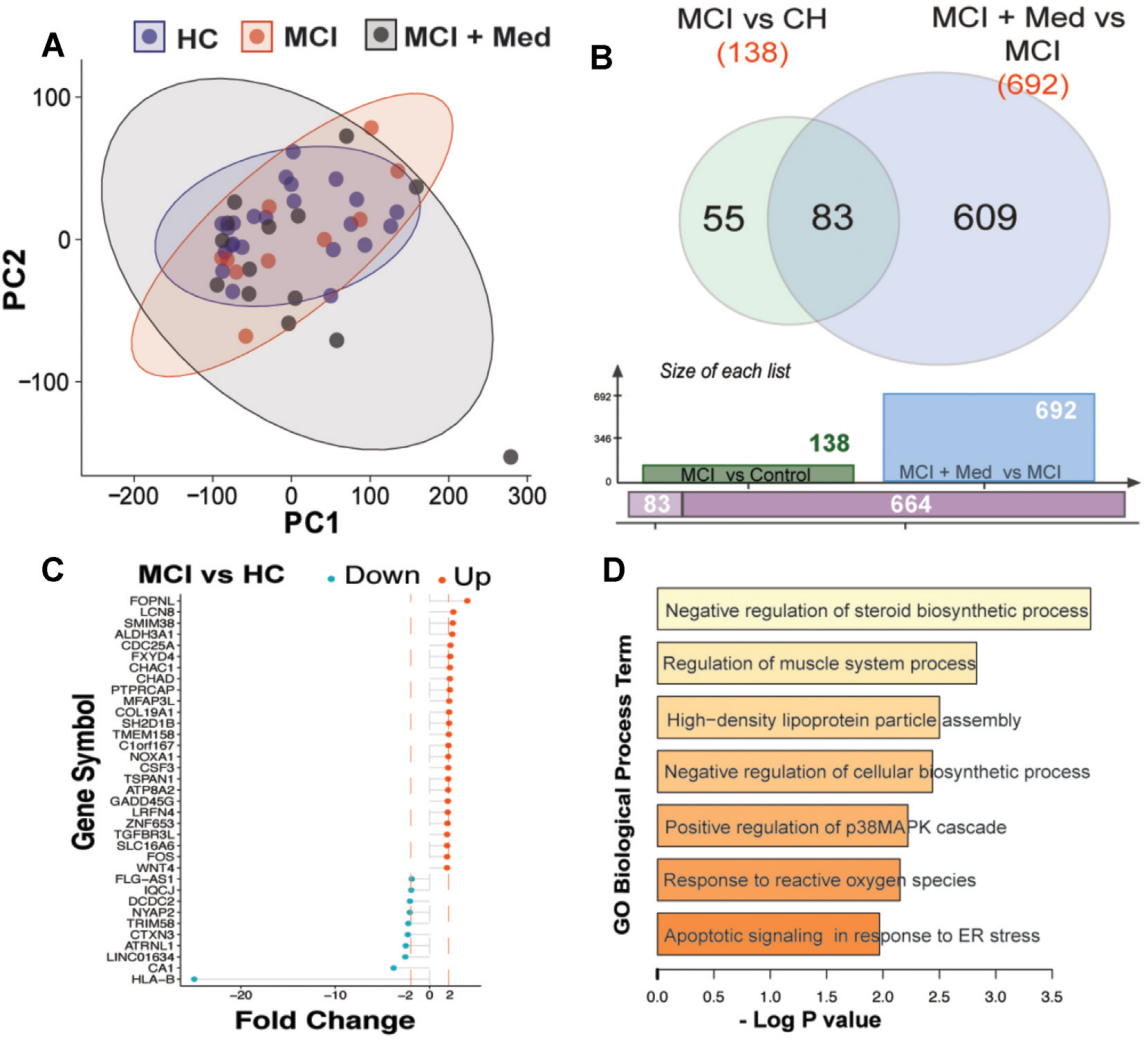
Global profiles and processes altered with MCI. Principal component analysis of of all expressed genes indicated that global profiles of genes were similar between the groups with the MCI + Med samples showing greater variability in expression (A). Comparisons between MCI and CH groups showed 138 differentially expressed genes, while donepezil-treatment revealed ∼692 differentially expressed genescompared to untreated MCI (B). Genes with a minimum 2-fold change are plotted in (C), and included HLA-B, which showed a 24-fold decrease in subjects with MCI relative to healthy controls. Gene ontology analysis showed that among genes influenced by MCI status, biological processes related to steroid biosynthesis, muscle metabolism, and lipoprotein assembly were significantly enriched (D).

We assessed protein expression via western blotting and gene expression via RNA sequencing with a focus on quantifying electron transport system related factors. in a subset of participants with sufficient available skeletal muscle samples (RNA; *n* = 47 and protein, *n* = 44). To assess expression, we specifically queried ∼80 genes known to be involved in the electron transport system, mitochondrial remodeling and biogenesis. We found a number of these genes to be downregulated in MCI individuals and recovered in donepezil-treated subjects (Figure 4). Although complex expression as assessed by western blot showed the same trends observed for the functional outcomes, these were not significantly different between groups (Figure S2).

### RNA Sequencing

As an exploratory analysis, gene expression profiling of skeletal muscle samples procured from biopsies from the three groups was carried out using RNA-sequencing. Principal component analysis of of all expressed genes indicated that global profiles of genes were similar between the groups with the MCI + Med samples showing greater variability in expression (Figure 4A). Comparisons between MCI and CH groups showed 138 differentially expressed genes (Figure 4B). Genes with a minimum 2-fold change are plotted in Figure 4C, and included major histocompatibility complex, class I, B (HLA-B), which showed a striking 24-fold decrease in subjects with MCI relative to healthy controls. Gene ontology analysis showed that among genes influenced by MCI status, biological processes related to steroid biosynthesis, muscle metabolism, and lipoprotein assembly were significantly enriched (Figure 4D).

**Figure 5.**
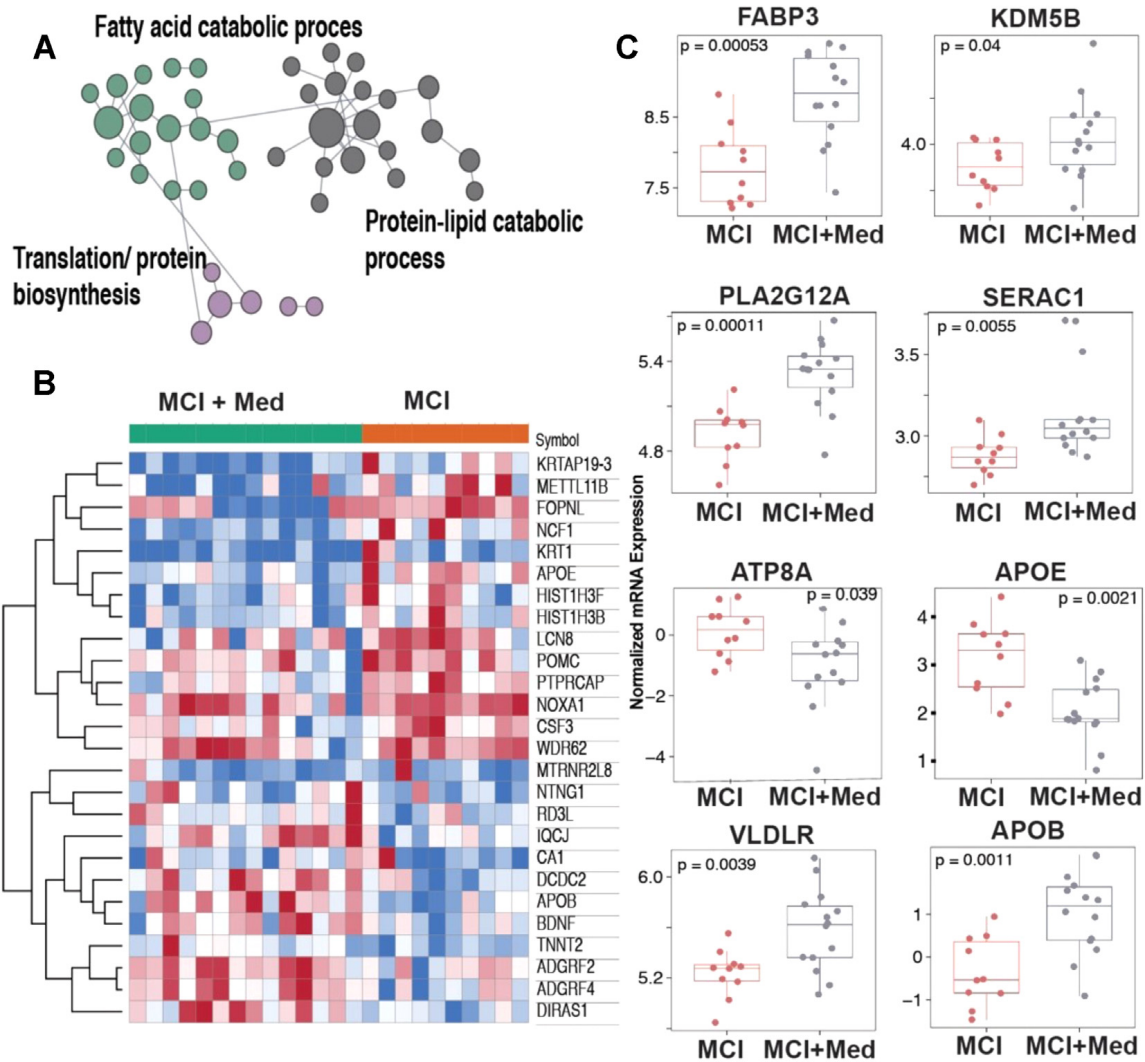
Processes differentially affected in untreated and donepezil treated MCI subjects. Tissue-specific network analysis using the HumanBase functional module enrichment releveled fatty acid metabolism, protein–lipid catabolic process and protein biosynthetic process terms to be enriched (A) with the top 25 differentially expressed genes in this comparison are presented as a heatmap (B). Specific query of genes related to lipid transport (GO:0006869). indicated that donepezil treatment significantly altered a number of lipid transporters including increased expression of FABP3, VLDLR, APOB, and SERAC1 among others (C).

Evaluation of donepezil treated MCI subjects compared to untreated counterparts (MCI + Med vs MCI) revealed a robust effect (∼692 differentially expressed genes) on muscle gene expression. Tissue-specific network analysis of these genes using the HumanBase functional module enrichment revealed fatty acid metabolism, protein–lipid catabolic process and protein biosynthetic process terms to be enriched (Figure 5A). The top 25 differentially expressed genes in this comparison are presented as a heatmap (Figure 5B). Notably, mRNA expression of APOE, NOXA1, and NCF1 were decreased in the MCI + med skeletal muscle compared to CH. Since lipid metabolic processes were significantly impacted by drug treatment, we specifically queried genes related to lipid transport (GO:0006869). These analyses indicate significant alteration in a number of lipid transporters including increased expression of FABP3, VLDLR, APOB, and SERAC1 among others in donepezil treated individuals (Figure 5C).

**Figure 6.**
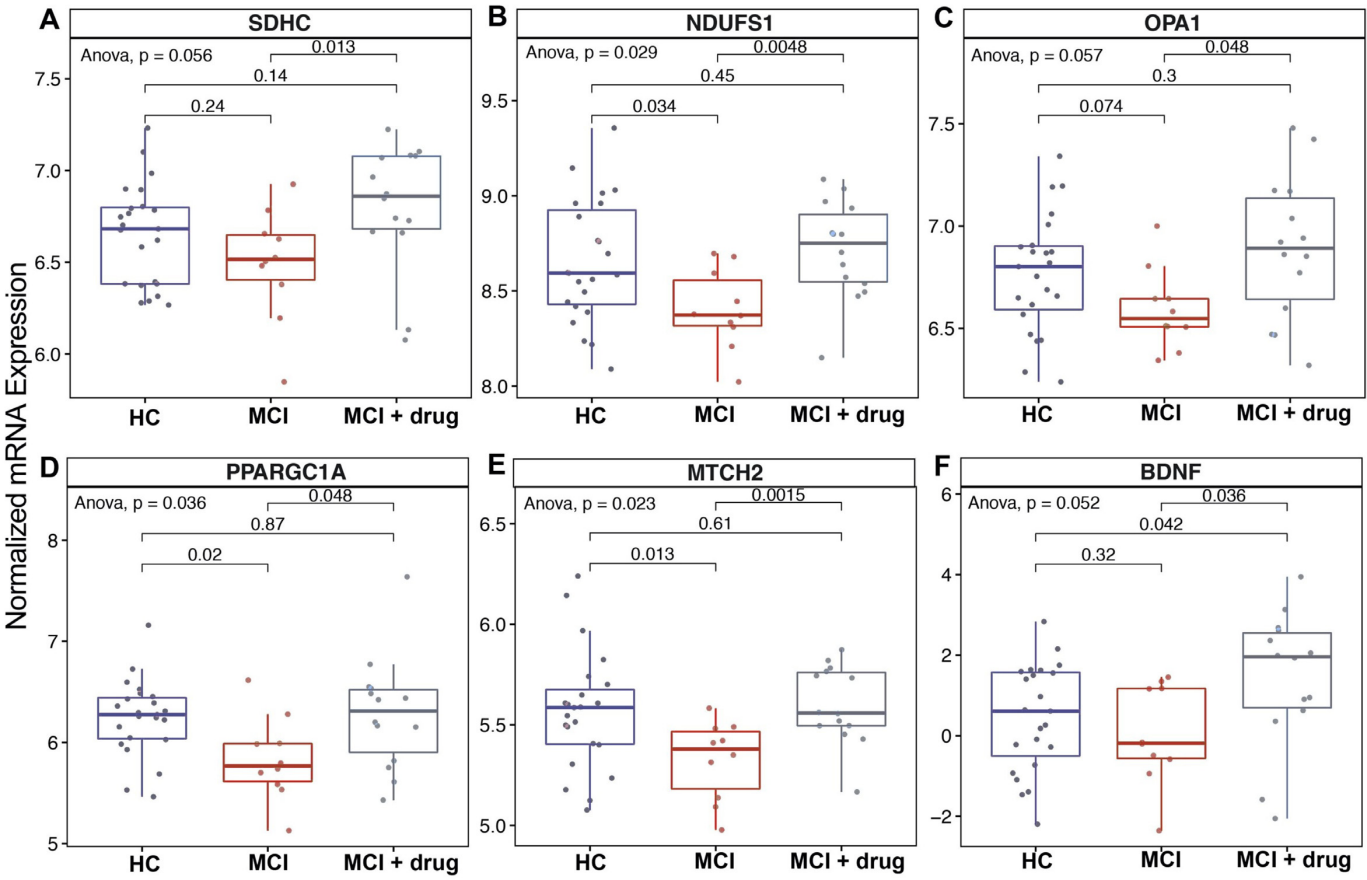
Expression of mitochondria-related genes across groups. Expression of genes related to mitochondrial function was changed in MCI muscle and in donepezil treated individuals. This included (A) a component of succinate dehydrogenase. SDHC), (B) the largest subunit of Complex 1 (NDUFS1), (C and E) mitochondrial fusion-related proteins (MTCH2 and OPA1), (D) the master regulator of mitochondrial biogenesis (PPARGC1A), and (F) brain derived neurotrophic factor (BDNF).

Lastly, given the large effects of MCI statusand medicaion on mitochondrial respiratory function in skeletal muscle, we focused analysis on mitochondrial genes. Succinate dehydrogenase (SDHC) expression was lower than CH and MCI + Med (Figure 6A). We found similar patterns for the largest subunit of Complex 1 (NDUFS1; Figure 6B), genes driving transcription of proteins known to mitochondrial fusion-related proteins (MTCH2 and OPA1; Figure 6C and E) and a master regulator of mitochondrial biogenesis and oxidative phosphorylation (PPARGC1A; Figure 6D). Finally, we also report that skeletal muscle gene expression of brain derived neurotrophic factor (BDNF) was lower in MCI than CH or MCI + Med (Figure 6F). All told, these data suggest a strong role of MCI and donepezil to influence skeletal muscle mitochondrial respiratory function through transcriptional regulation.

### Adverse Events

There were six adverse events possibly or definitely related to the GXT or muscle biopsy: two mild, and four moderate in severity. Mild adverse events possibly or probably related to participation included abnormal screening labs (1) and prolonged muscle soreness after the muscle biopsy (1). Moderate severity adverse events included heart rhythm abnormalities during fitness testing (3) and fainting after muscle biopsy (1).

## Discussion

We report here the first ex vivo assessment of skeletal muscle mitochondrial function in MCI, the earliest clinical stage of AD. Our primary finding is that unmedicated MCI participants display reduced skeletal muscle mitochondrial respiratory capacity (Figure 1) and poorer coupling control (Figure 2) compared to CH older adults. This suggests that prior findings of reduced insulin sensitivity and aerobic capacity in AD^2,3^ may be partly driven by changes in skeletal muscle mitochondrial function. MCI subjects who are treated with AD medication (primarily the acetylcholinesterase inhibitor donepezil) displayed mitochondrial respiration values in line with CH subjects. Moreover, Donepezil treated MCI patients had the biggest differences in skeletal muscle gene expression profiles compared to CH and MCI untreated groups. Donepezil increased the gene expression of spedific transcriptional regulators of mitochondria that were lower in MCI untreated muscle. Donepezil significantly reduces the rate of cognitive decline in early AD and our results suggest it may also mediate protection against reductions in skeletal muscle mitochondrial respiratory capacity. However, these cross-sectional analysis require future prospective (pre and posttreatment) studies to confirm if reduced skeletal muscle mitochondrial function plays an important role in MCI and ad, and if a commonly used ad medication can indeed impact skeletal muscle mitochondrial function in MCI or in other aging-associated disease states.

There is growing evidence for bioenergetic deficits playing a primary role in ad pathology. Cerebral hypometabolism, a marker of reduced energy metabolism, is an early ad biomarker^34,35^ and occurs first in highly metabolic brain regions.^36,37^ While the bulk of energy in the brain iderives via oxidative phosphorylation in mitochondria, it remains difficult to assess cerebral mitochondrial function directly. However, there is evidence mitochondrial function may be compromised systemically in early ad.^1,38,39^ It has also been shown that reactive oxygen species produced in mitochondria enhance Aβ production, which can deposit within mitochondria resulting in further deficits.^39,40^ad-pertinent transgenic animal models also show mitochondrial deficits. Mice transgenic for human *APOE4* display mitochondrial dysfunction in neurons,^41^ and both the double and triple transgenic ad mouse models^42,43^ display marked mitochondrial dysfunction in skeletal muscle.^43,44^ Our findings of lower skeletal muscle mitochondrial respiration in MCI participants compared to cognitively healthy older adults support a growing body of evidence associating bioenergetic alterations with cognitive decline. To our knowledge this is the first study to directly characterize an ex vivo skeletal muscle mitochondrial function difference in MCI compared to CH.

Preclinical evidence also supports our observations of enhanced mitochondrial function in medication treated MCI subjects. In our study, all 15 subjects in the MCI + med group were receiving the cholinesterase inhibitor donepezil. In transgenic AD mice, donepezil ameliorated cognitive deficits independent of acetylcholinesterase inhibition and decreased accumulation of Aβ in brain mitochondria.^45^ Pretreatment of rat brain mitochondria with donepezil also decreases mitochondrial Aβ accumulation after oligomeric Aβ exposure and mitigates an Aβ-related decline in ATP production, further suggesting protection against Aβ-induced deficits.^45^ Donepezil elicits antiamnestic effects and protection against Aβ-induced neurodegeneration when both are administered intracerebroventricularly in mice.^46^ It is possible that some benefits of donepezil are mediated through the sigma 1 receptor, a chaperone that localizes to mitochondria associated endoplasmic reticulum membranes.^47^ This is of interest because endoplasmic reticulum–mitochondria communication may be a factor in ad-related metabolic disturbances and lower mitochondrial respiration.^48^ Increasing cholinergic signaling, the intended consequence of cholinesterase inhibitor therapy, also induces mitochondrial biogenesis in both primary cultures of mouse hippocampal neurons and in hippocampal tissue, an effect linked to induction of AMP-activated protein kinase (AMPK) and peroxisome proliferator-activated receptor gamma coactivator 1-alpha (PGC1α).^49^ Notably, we saw that MCI patients taking Donepezil had increased expression of PGC1 α in skeletal muscle. Other cholinesterase inhibitors, or simply enhancing cholinergic tone, produce benefits in cell studies, including improved mitochondrial function, decreased endoplasmic reticulum stress, and prevention of amyloid-beta induced apoptosis.^50,51^

It should be noted that the NMDA receptor antagonist memantine was concomitantly used with donepezil in 5 MCI + med subjects. Memantine also stimulates mitochondrial function^52^ and upregulates autophagy, enhancing clearance of damaged mitochondria from cultured neuronal cells.^53^ Thus it is likely that mitochondria-related effects in response to ad medication use may be of clinical relevance. In humans, the symptomatic benefits of donepezil have historically been attributed to effects on acetylcholine neurotransmission,^54^ and it is debated as to whether cholinesterase inhibitors significantly modify the course of ad. Some degree of neuroprotection has been proposed with donepezil,^55,56^ and donepezil treatment associated with slowed hippocampal atrophy.^57^ A meta-analysis of over 40 randomized clinical trials involving cholinesterase inhibitors suggests a reduction in mortality, supporting a potential disease-modifying effect.^58^ Positive long term results have led investigators to suggest that cholinesterase inhibitors improve redox balance^59^ and oxidative capacity.^60^ This is supported by the main findings in our study, which show a decline in skeletal muscle mitochondrial respiratory capacity in unmedicated MCI subjects but not in the MCI + med group. The novelty in our findings is that commonly used ad medication treatment was associated with improved mitochondrial outcomes in skeletal muscle. However, a prospective treatment study is needed to determine if the drug truly improves mitochondrial function in skeletal muscle. Despite the MCI + med group showing improved state 3 respiration, the treated group still showed reduced coupling and leak control ratio in line with MCI not taking medication. These impaired coupling values may be due in part to increased rates of basal proton leak.^61^ Overall, this suggests that mitochondrial impairment exists in MCI, and while donepezil treatment may induce compensatory pathways.

We examined the relationship between cardiorespiratory fitness and ADP-stimulated mitochondrial respiration in individuals who met established ACSM criteria for a maximal exercise test ^62^ (VO_2max_; n = 44). We observed a significant, positive linear relationship between aerobic capacity and ADP-stimulated mitochondrial respiration in both CH and MCI groups (Figure 3). This is consistent with prior work showing that skeletal muscle mitochondrial outcomes (content and function) are associated with whole-body aerobic capacity in cognitively healthy individuals throughout the lifespan.^63–65^ In contrast, there was no relationship between aerobic capacity and mitochondrial respiration within medication treated MCI subjects. Interestingly, mitochondrial respiration was not related to aerobic capacity in any group when we did not use ACSM criteria for a valid exercise test (VO_2peak_ instead of VO_2max_)—an important consideration for studies examining the relationship between cellular respiration and whole-body fitness.

We notably found that both unmedicated MCI and MCI + med subjects exhibited lower fasting RER values during quiet rest prior to the GXT. Both groups of MCI subjects appear to be more primed to use fatty acid substrates compared to cognitively healthy elderly adults, who exhibit an RER with a greater reliance on carbohydrate use. Somewhat surprisingly, we did not observe any differences in the plasma lipid profile or fasting markers of insulin resistance between groups. This may be due to a variety of factors, including the early disease stage, the high degree of physical activity in all the subjects, the cross-sectional nature of the study, or other factors known to affect lipid homeostasis, such as genetics. Nevertheless, this indicates that the mitochondrial bioenergetic differences can exist outside of or even precede whole-body insulin resistance and/or dyslipidemia.

To expand upon our functional outcome measures, we also performed exploratory analyses using next-generation RNAseq to explore gene expression profiles in muscle amongst our groups. Given the observed effects on mitochondrial function and metabolic function, we postulated that genes involved in these processes would be differentially regulated between groups. Our findings indicate that early stage cognitive impairment is associated with systemic changes in biological processes related to steroid biosynthesis, muscle metabolism and lipoprotein assembly. Interestingly, we also observed a striking downregulation of HLA-B, part of the major histocompatibility complex that plays a critical role in immune response,^66^ in MCI muscle compared to CH.

We anticipated that we would observe differences in skeletal muscle electron transport system gene and protein expression between groups, as prior work suggests cytochrome oxidase deficits in MCI and ad,^39,67^ and a recent meta-analysis has shown complex IV deficits in ad brain.^68^ Indeed, when we explored differences in electron transport system related genes, we observed that expression of several important genes were downregulated in MCI but comparable to the CH in the donepezil-treated group. These included the master regulator of mitochondrial biogenesis (PPARGC1A), the largest subunit of Complex 1 (NDUFS1), a component of succinate dehydrogenase (SDHC; *P* = .056) and mitochondrial fusion-related proteins (MTCH2 and OPA1). We also observed similar trends when assessing ETS protein levels (*P* = .06 for Complex IV), although these difference did not reach significance, which could be due to the small size of the MCI group.

In donepezil-treated cognitively impaired individuals, there was differential expression of genes related to fatty acid metabolism, protein–lipid catabolic process, and protein biosynthetic processes in muscle. This included notable decreases in both NADPH oxidase activator 1 (NOXA1) and neutrophil cytosolic factor 1 (NCF1), genes that promote reactive oxygen species production,^69,70^ as well as APOE, a key effector of lipid metabolism and transport.^71^ Since lipid metabolic processes were significantly impacted by medication treatment, we specifically queried genes related to lipid transport (GO:0006869). These analyses indicated that individuals receiving donepezil (MCI + med) exhibited significant alterations in a number of lipid transporters and genes linked to mitochondrial function, including increased expression of FABP3, SERAC1, VLDLR, and APOB, among others. Broadly, these results suggests that MCI status and drug treatment significantly affect gene expression in skeletal muscle. Overall, the principle component anlalysis revealed that the biggest group differences in gene expression occurred between the MCI + med vs. MCI group suggesting that donepezil may have a powerful effect on transcriptional programming in skeletal muscle, an effect which certainly needs further investigation.

A major strength of this study is the robust ex vivo measures of mitochondrial respiration in skeletal muscle biopsy tissue, which is novel and has never been characterized in human subjects with MCI. However, there are limitations. Muscle biopsies are somewhat invasive and our sample size is limited. This restricted our ability to parse out potential interaction relationships between diagnostic, genetic, sex, and anthropometric characteristics, especially in terms of medication use and dose. Future work should further investigate the effects of these important factors, as well as the relationship of bioenergetics with functional activity outcomes. Our study also focused on MCI diagnosed in a clinical setting from community-based providers. Although MCI can be due to different causes, longitudinal studies suggest that a high percentage of MCI is due to AD. Our plasma assessments that indicate greater neuropathology (Aβ 42:40) and neurodegeneration (NFL) in MCI subjects, which supports that our participants are enriched in MCI due to ad. Nonetheless, additional work on MCI subjects with further characterization, such as harmonized neuroimaging measures, or tissue analyses from individuals prior to and after donepezil treatment is needed to confirm the findings of this cross-sectional study. Our clinically-relevant findings suggest that there is mitochondrial change in skeletal muscle associated with a disease that occurs in the brain. Furthermore, it suggests a potential new mechanism by which donepezil may directly or indirectly impart benefit in ad-related cognitive decline. Finally, while a decrease in respiration in MCI compared to the cognitively healthy and medication treated groups is putatively pathological, further work is needed to determine if this is a negative effect, and longitudinal studies should be performed to whether changes in whole body mitochondrial bioenergetics precede early cognitive decline. Additional work is also needed to clarify the degree to which bioenergetic change tracks with neuropathological markers of decline over time.

In conclusion, we show reduced mitochondrial respiration in skeletal muscle, a metabolic tissue that is critical for strength, movement, and metabolism particularly in aging, during the earliest stages of ad-related cognitive decline; and effects that appear to be affected by the commonly used ad drug donepezil. RNA sequencing relevaled interesting effects on genes involved in mitochondrial function and muscle metabolism. This work provides additional evidence of systemic mitochondrial dysfunction in MCI and AD.

## Supplementary Material

zqab045_Supplementary_Data
