# Supplementary material for: Mild Cognitive Impairment and Donepezil Impact Mitochondrial Respiratory Capacity in Skeletal Muscle
Source: Function (Oxf). 2021 Sep 2;2(6):zqab045. doi: 10.1093/function/zqab045 (PMC8515006; doi:10.1093/function/zqab045)
Supplement: zqab045_Supplementary_Data [file zqab045_supplementary_data.docx]

**Supplementary Data**

**Supplementary Figure 1**


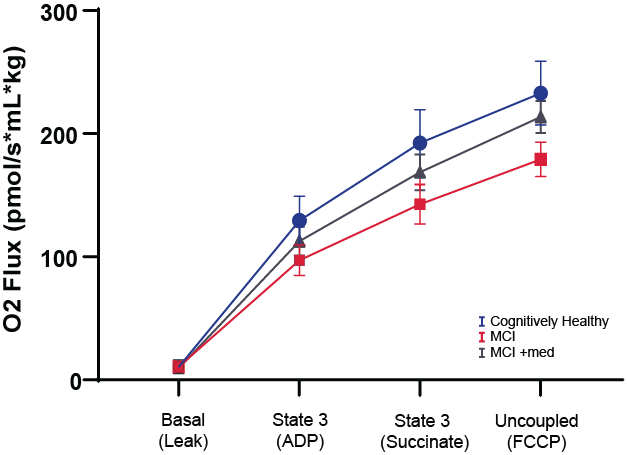


**Supplementary Figure 1.** Carbohydrate (pyruvate/glutatmate/malate) stimulated skeletal muscle mitochondrial respiration trended towards differences in the hypothesized direction, but did not reach significance across the various conditions tested.

**Supplementary Figure 2**

**Skeletal muscle protein expression of mitochondrial complexes.**


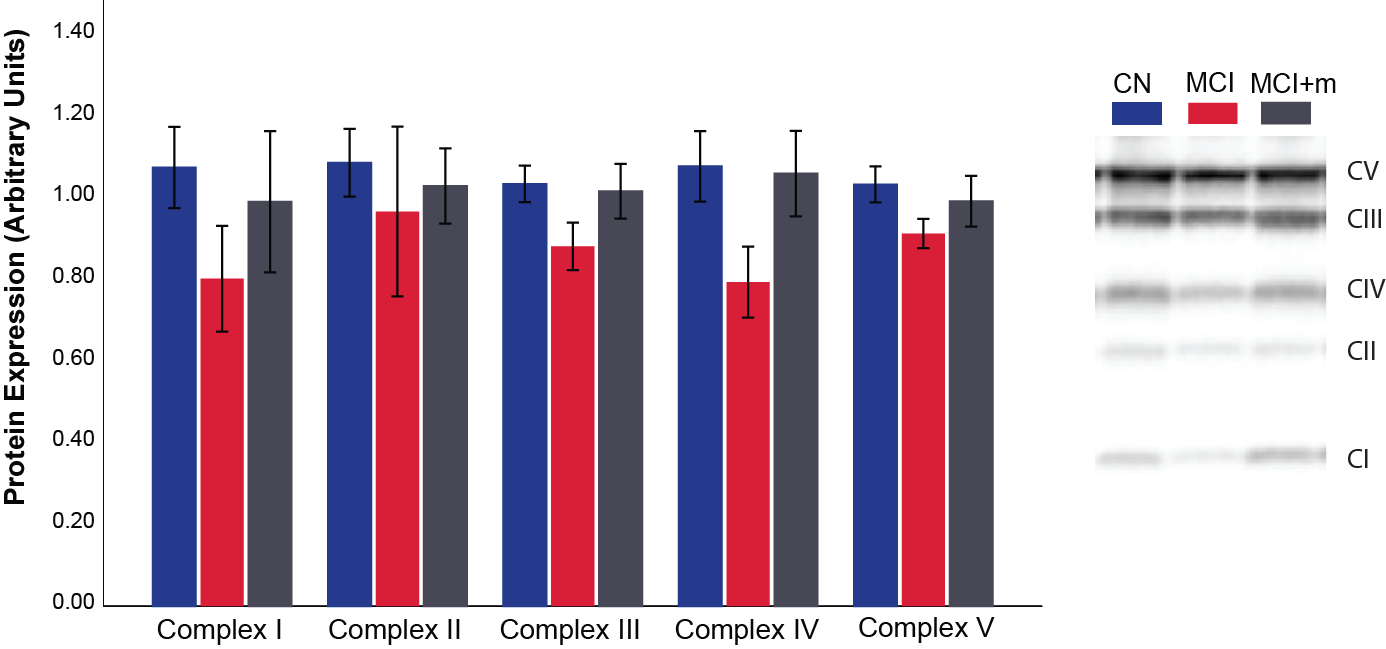


**Supplementary Figure 2.** Untreated MCI individuals exhibited a consistent but nonsignificant trend for lower protein expression of mitochondrial complex proteins in skeletal muscle**.** Group differences were assessed using ANCOVA, adjusting for covariates. These analyses were performed on a subset of individuals (n=44) for whom sufficient muscle sample was available (CH n=21, MCI n=10, MCI+med n=14). Data is shown as means ± SE.
